# Application of deformity principles in the management of spinal neoplasms: A Primer

**DOI:** 10.1016/j.xnsj.2025.100779

**Published:** 2025-08-05

**Authors:** Zach Pennington, Joseph H Schwab, Sheng-fu Larry Lo, C. Rory Goodwin, Matthew L Goodwin, Matthew Colman, Raphaële Charest-Morin, Nicolas Dea, Daniel Lubelski, Ali Ozturk, Jacob M. Buchowski, Wende Gibbs, Wesley Hsu, Ajit Krishnaney, Ilya Laufer, Mohamed Macki, Addisu Mesfin, Ganesh Shankar, Dan Tobert, John Shin, Andrew Platt, Daniel M Sciubba

**Affiliations:** a Department of Neurosurgery, Mayo Clinic, 200 1st Street SW, Rochester, MN 55905, United States; b Department of Orthopedics, Cedars-Sinai Medical Center, 8700 Beverly Blvd, Los Angeles, CA 90048, United States; c Department of Neurosurgery, Zucker School of Medicine at Hofstra, Long Island Jewish Medical Center and North Shore University Hospital, Northwell Health, 300 Community Dr., 9 Tower, Manhasset, NY 11030, United States; d Department of Neurosurgery, Duke University School of Medicine, 10 Duke Medicine Cir, Durham, NC 27710, United States; e Department of Orthopedic Surgery, Washington University School of Medicine, 660 S Euclid Ave, St Louis, MO 63110, United States; f Midwest Orthopedics, Rush University School of Medicine, 1620 W Harrison St, Chicago, IL 60612, United States; g Department of Orthopedics, University of British Columbia, 2775 Laurel Street, Vancouver, British Columbia V5Z 1M9, Canada; h Department of Neurosurgery, Johns Hopkins University School of Medicine, 1800 Orleans St, Baltimore, MD 21287, United States; i Department of Neurosurgery, University of Pennsylvania School of Medicine, 3400 Civic Center BLvd, Philadelphia, PA 19104, United States; j Department of Radiology, Barrow Neurological Institute, 2910 North Third Avenue, Phoenix, AZ 85013, United States; k Department of Neurosurgery, Wake Forest University School of Medicine, 475 Vine St, Winston Salem, NC 27101, United States; l Department of Neurosurgery, Cleveland Clinic, 9500 Euclid Ave, Cleveland, OH 44195, United States; m Department of Neurosurgery, New York University School of Medicine, 550 First Avenue, New York, NY 10016, United States; n Department of Orthopedics, MedStar Health, 3800 Reservoir Rd NW, Washington DC 20007, United States; o Department of Neurosurgery, Massachusetts General Hospital, 55 Fruit Street, Boston, MA 02114, United States; p Department of Orthopedic Surgery, Massachusetts General Hospital, 55 Fruit Street, Boston, MA 02114, United States; q Department of Neurological Surgery, Kaiser Permanente – Fontana Medical Center, 9961 Sierra Ave, Fontana, CA 92335, United States

**Keywords:** Spine tumor, Spinal deformity, Radiotherapy, Carbon fiber, Proton beam radiotherapy, Patient-reported outcomes, Spinal alignment

## Abstract

**Background:**

With advances in surgical techniques, radiation, and systemic therapy, prognoses and quality of life have improved amongst patients with primary and metastatic vertebral column tumors. Sagittal deformity is known to have an adverse impact on patient quality of life but has been largely ignored in this study population.

**Methods:**

A comprehensive literature review was conducted, focusing on articles germane to the study of spinal deformity in the context of oncologic disease. Articles included those focusing on bone health, the association of spinal deformity with oncologic spine disease, and both pelvic and anterior column reconstruction in patients treated for primary tumors.

**Results:**

Little to date has focused specifically on the management of spinal deformity in the context of spinal tumors. However, it is known that tumor involvement of the vertebral column is associated with poorer screw purchase, which can be further worsened by radiotherapy. Instrumentation techniques that seek to address underlying deformity must also balance the need for radiographic follow-up, which is improved with novel carbon fiber-reinforced polyetheretherketone implants, and the need for intraoperative contouring. Last, residual deformity is associated with poorer patient reported outcomes and increased mechanical complications in adult spinal deformity, but better study within the spinal oncology population is merited.

**Conclusion:**

The potential negative impact of spinal deformity on patient quality of life in the spinal oncology population is now better appreciated amongst spinal oncologists, but studies have been limited to date. Further investigation is merited as survival outcomes continue to improve.

## Background

Spine oncology encompasses both benign and malignant entities that localize to both the vertebral column and the spinal cord and meninges. Conventionally spinal oncology has focused on local control, maintenance of neurologic function, stabilization, and overall patient survival. However, as cancer prognoses have improved over the past 10 to 15 years [1], there has been increased recognition of the correlation between patient quality of life and overall spinal alignment (specifically sagittal balance) [2,3]. Surgeons are therefore also increasingly considering spinal alignment in these patients. Tumor surgery represents a chance to opportunistically correct spinal deformity, as resection often requires vertebral column resection—a special 3 column osteotomy that affords the chance for significant sagittal plane correction. The present review by the North American Spine Society Section on Spinal Oncology therefore serves as a primer on modern deformity principles for spinal oncology surgeons and focuses on the application of deformity principles to the treatment of spine tumor patients.

## Overview of spinal deformity and patient quality of life

### Alignment and patient-reported outcomes

Work by Glassman et al. [2,4], noting sagittal, not coronal balance best predicts clinical symptoms in adult spinal deformity (ASD). In the follow-up study [2], they found sagittal balance positive correlated with both SF-12 physical health status and Oswestry Disability Index. Multiple other studies have looked at alignment and both disease-specific and general health PROs [5,6]. In general these have also found greater sagittal imbalance to correlate with worse pain and quality of life [6], though there remains no consensus regarding which sagittal alignment parameter best predicts PRO outcomes. One recent study by Zhang et al. [6] suggested SVA was most highly correlated with outcomes but others have suggested that pelvic incidence (PI)—lumbar lordosis (LL) mismatch (PI-LL) [[7], [8], [9], [10]] or pelvic tilt (PT) [9,11] may be an effective predictors. Evidence remains mixed though [12,13]. Within the spine tumor population such literature is even more limited. However, Massier et al. [14] noted that the Global Alignment and Proportion (GAP) score correlated with patient-reported physical function scores following *en bloc* resection of mobile spine tumors even after correcting for local disease recurrence.

### Summary of modern deformity parameters

Much of ASD surgery focuses on sagittal balance—a description of the body’s “tendency” to try and maintain horizontal gaze and keep the center of gravity of the head and shoulder girdle above the pelvis. This has also been described as the “cone of economy” by Dubousset [15], who described the increased musculoskeletal strain that occurs when the patient’s posture is displaced too far from the upright position. Patients demonstrate [often subconscious] compensatory mechanisms to maintain sagittal balance, including thoracic spine hypokyphosis, pelvic retroversion, and knee flexion. As a result, patients can present with back, anterior thigh, and buttock pain that worsens throughout the day due to chronic postural muscle activation. Radiographically, the patient’s pelvic retroversion is manifest as an increased PT or a flattening of the iliac crests on posterior-anterior standing radiographs. Head-to-toe radiographs are imperative to fully assess the patient’s spinal deformity and compensatory mechanisms. For some spine tumor patients this may be infeasible owing to pain, neurological deficits, or extremity disease that prohibits them from standing. Supine films may be a reasonable alternative for these patients; however, it should be noted that in most oncology patients, the deformity is mobile, not fixed, and so the deformity may be underestimated on supine images.

**Fig. 1:**
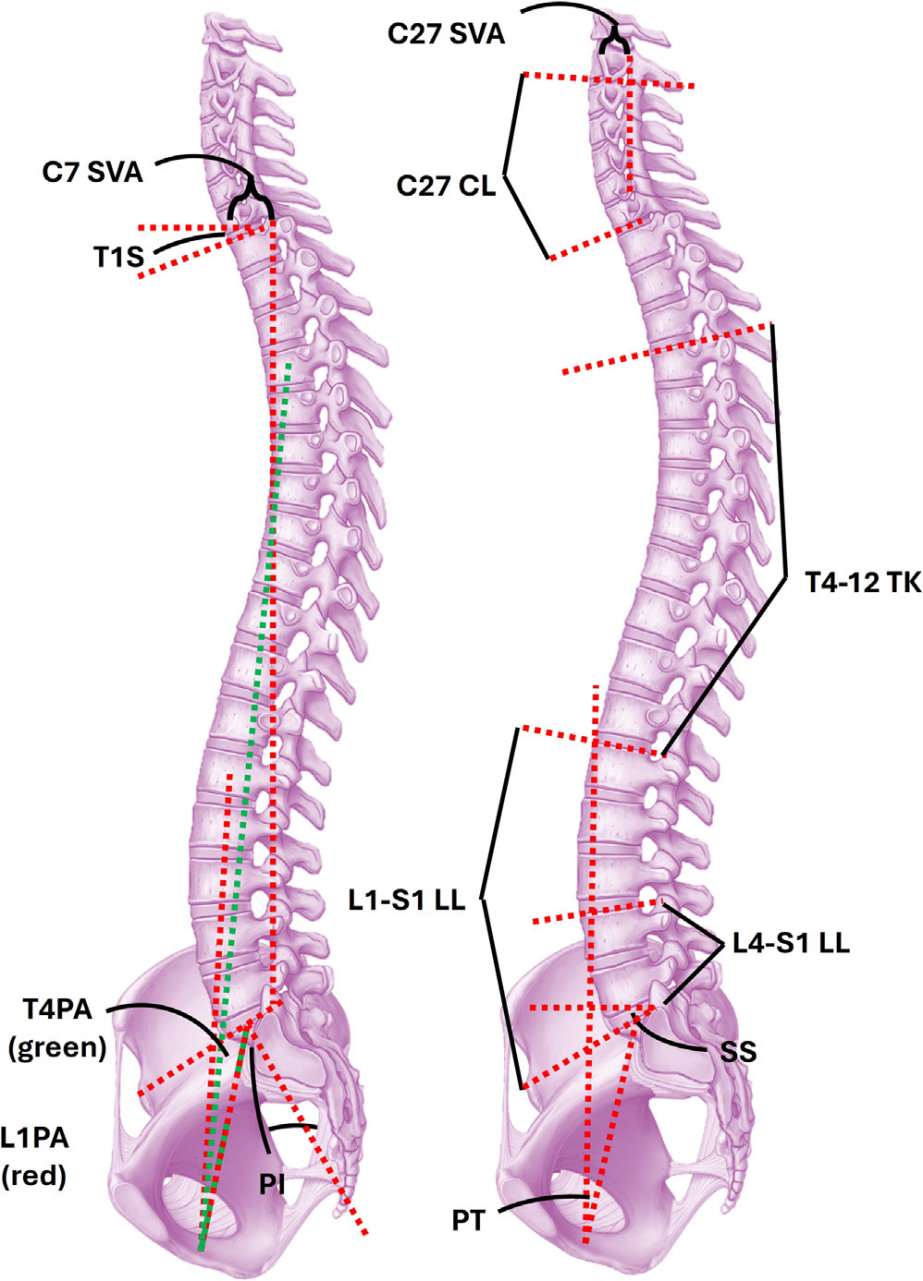
Illustration of radiographic parameters employed in modern deformity surgery. Global sagittal balance is commonly assessed with the C7 sagittal vertical axis (C7-SVA) with a value of ≤4 cm being considered normal and values >4 cm being correlated with poorer patient quality-of-life. An analogous parameter exists solely for the cervical spine (C2-7 SVA) with a normative value of <4 cm. In adult spinal deformity (ASD), achieving lumbopelvic harmony is a cornerstone of surgical planning and is encapsulated by the pelvic incidence (PI) – lumbar lordosis (LL) mismatch (PI-LL). Work by Roussouly and colleagues has aimed to describe the distribution of the lordotic curvature within the lumbar spine, but as a rule of thumb, approximately 2/3 of the total lumbar lordosis (L1-S1) is found in the lower 2 segments, the L4-S1 LL. Lumbopelvic harmony is achieved when PI-LL is <10°, where the pelvic incidence describes the relationship of the sacrum to the remainder of the bony pelvis. It is conventionally held to be fixed but has increasingly been noted to change with position and following long-segment lumbar fusion. A similar relationship to the PI-LL mismatch is described for the cervical spine between the cervical lordosis (C27 CL) and the T1 slope (T1S). Curvature within the spine is described using the cervical (CL), thoracic (T4-12 TK), and lumbar curvature (L1-S1 LL). Global balance has also increasingly been described using the L1-pelvic angle (L1PA) and T4-pelvis angle (T4PA), which show greater interobserver reliability compared to more conventional measures, such as C7 SVA. Patients with sagittal imbalance may show pelvic retroversion, which appears as a decreased pelvic tilt (PT); there is often a commensurate flattening of the sacral slope.

Fig. 1 provides an overview of commonly used sagittal balance parameters; these are also in other reviews on ASD [16]. Global balance can be described by the C7 sagittal vertical axis (C7-SVA)—the horizontal distance between the C7 centroid and the posterior superior corner of the S1 endplate – attempts to describe whether the shoulder girdle falls over the sacrum; normal is ≤4 cm [17] C7 SVA does not account for compensatory mechanisms and global tilt (GT), L1-pelvic angle (L1PA), and T4-pelvic angle (T4PA) are other metrics that attempt to encapsulate global balance while eliminating the influence of pelvic retroversion and knee flexion. T4PA is increasingly popular due to its high interobserver reliability [18] and ability to be assessed intraoperatively on prone radiographs. Cervical spine alignment can similarly be quantified using the C2-7 SVA, which is designed to assess whether the patient’s skull sits over the C7 body—the point of transition between the cervical and thoracolumbar spine. Chin-brow vertical angle (CBVA) is also important in cervical deformity correction and describes the patient’s horizontal gaze [19,20]. A patient’s compensatory mechanisms are often attempts to maintain horizontal gaze.

Global balance is itself a sum of the regional balance of the thoracic and lumbar spines, and the degree to which they show lumbopelvic harmony. The normal curvatures of the thoracic and lumbar spine are kyphosis and lordosis, respectively, though there can be a loss of thoracic kyphosis (TK) in the setting of global sagittal imbalance. In the context of spinal malignancy, there is often anterior column collapse with focal hyperkyphosis. Roussouly [21] also further analyzed the lumbar spine regional curvature, noting interpersonal variation in the apex of the lumbar curvature. As a rule of thumb though, most patients have two-thirds of their LL (usually 35–40°) in the 2 caudal most segments (L4-S1) [22].

When correcting sagittal plane deformity, surgeons seek to match the LL and PI—a mathematical description of how the sagittal relationship between the mobile spine and pelvis. It is conventionally held to be fixed, though it is increasingly recognized that it changes slightly with position and can even change after pelvic fixation [23]. Ideal PI-LL mismatch is generally accepted as <10° and residual PI-LL mismatch is associated with increased risk of implant failure and adjacent segment disease in patient undergoing *en bloc* vertebral column tumor resection [24]. L1PA also attempts to encapsulate lumbopelvic harmony. Defined as PI/2 – 21°, it may better predict hardware failure than PI-LL [25] and strongly correlates with T4PA [26], though results are mixed and L1PA may not effectively predict PROs [27].

## Considerations for spinal realignment

### Bone quality

Bone quality and implant selection are key to successful spinal realignment as the ability to perform corrective maneuvers intraoperatively is limited by screw purchase strength. Conventionally, bone quality is assessed with dual-energy X-ray absorptiometry (DXA), which determines the bone mineral density (BMD) based upon the absorption of X-rays projected at the hip, femur, lumbar spine, or wrist [28]. The calculated BMD is converted to a T-score, classifying their BMD relative to young, sex-matched controls: a T-score >−1 is normal, one between −1 and −2.5 is osteopenia, and *T*≤−2.5 is osteoporotic. Low BMD (T-score <−1) has been correlated with a significant increase in vertebral compression fracture risk [29], a surrogate marker for anterior column structural incompetence under axial loading. Additionally, low BMD is highly correlated (*r*=0.93) with screw pullout strength [30] and construct fatigue resistance, with osteoporotic bone tolerating 55% fewer cycles to failure relative to normal BMD *in vitro* [31]. However, DXA imperfectly captures cancellous bone volume and strength [32,33], and may be inaccurate in patients with compression fractures, obesity, or degenerative spine changes [28,34].

Alternatives to DXA of increasing interest are the measurement of Hounsfield Units (HU) on computed tomography (CT) scans and the Vertebral Bone Quality (VBQ) score on magnetic resonance imaging (MRI) [28,[35], [36], [37], [38]]. HU-based bone strength measures operate on the same principle as DXA with higher bone densities causing greater X-ray absorption and higher HU values. *In vitro* testing has shown strong correlation to both vertebral body ultimate load and stress on compression [39]. Additionally, HU on CT have been correlated with biomechanical CT-based measures of vertebral whole-bone strength and trabecular BMD [40]. Unlike DXA, it can also be measured using imaging routinely obtained for spine tumor patients for example, the CT chest/abdomen/pelvis obtained as part of systemic disease staging. However, this is with the caveat that these staging images commonly use intravenous contrast, which has a small, but significant impact on HU measures [41]. Additionally, CT-based HU are measured for the bones of interest—those undergoing instrumentation—as compared to DXA, which is usually performed using the appendicular skeleton. For the lumbar spine, HU values of <110 and <160 are considered to be cutoffs for osteoporosis, and osteopenia, respectively, as they correlate with accepted quantitative CT measures of <80mg/cm³ and <120mg/cm³, respectively [42,43]. Similarly, HU values of <341 and <327 have been suggested as markers of osteopenia and osteoporosis in the cervical spine [44], and HU values of <153 and <231 have been suggested as markers for osteoporosis [45] and osteopenia [46] in the mid-thoracic spine [45]. Several studies have shown HU to predict mechanical complications following instrumented spine surgery [35,47].

VBQ is another alternative and has been shown to predict mechanical complications following spinal fusion [[48], [49], [50]]. It measures the T1 signal intensity of the bone marrow at multiple levels. Normalization with the signal intensity of adjacent cerebrospinal fluid is intended to overcome differences in MRI field strength and scanner type, though it does not fully overcome these differences which are based on echo time [38]. It operates on the assumption that cancellous bone trabecular loss is associated with fatty infiltration which produces increased T1 signal [51]. However, other materials, such as blood product can cause increased T1 signal and tumor-infiltrated vertebra can show increased or decreased T1 signal depending upon the underlying pathology [52]. Consequently, it may not accurately capture composition and bone quality of the tumor-infiltrated bone, which will affect the biomechanical properties. Direct comparisons of HU and VBQ have demonstrated HU to be more predictive of the occurrence of complications in both the ASD [35] and spine oncology populations [36]. Our preference therefore is to employ HU measurements.

An additional consideration in the spinal metastasis population is the Spinal Instability Neoplastic Score (SINS) [53]. It considers the degree of vertebral body involvement/collapse, posterior element involvement, presence of associated focal deformity, lesion quality (osteolytic, osteoblastic, mixed), the presence of pain (oncologic, mechanical or none), and lesion location along the spine axis. Though developed for spinal metastases, the principles SINS encapsulates include the significant load-bearing performed by the anterior column, the presence of greater stresses at junctional levels, and the crucial contribution of the posterior tension band to maintaining alignment. Despite its utility for identifying underlying instability, SINS does not incorporate assessments of spinal alignment and is likely inadequate for assessing deformity and alignment targets in patients undergoing tumor surgery with concomitant deformity correction.

### Implant selection

Implant selection includes selection of longitudinal elements (eg, rods, tethers), anchors employed (eg, screws, hooks), and anterior column support (eg, corpectomy cage). This includes evaluating implant stiffness for maintaining correction postoperatively, use of a multirod constructs for high-stress regions (eg, 3-column osteotomy or corpectomy levels), screw pitch (eg, corticocancellous vs cortical vs pure cancellous thread pitch), rod diameter, and the use of a “soft stop” to prophylax against adjacent segment pathology. Spinal oncology cases must additionally consider the need for postoperative radiotherapy and serial radiographic follow-up for lesion recurrence/progression. Of the conventional implant materials, titanium alloy rods create less artifact than cobalt chrome rods [54], allowing for improved surveillance and more accurate radiotherapy planning [55]. They are also less stiff, which can be advantageous when trying to avoid screw pullout from compromised bone.

Anterior column reconstruction with metal implants can further complicate postoperative radiotherapy [56], as the resultant beam scatter increases spinal cord radiation dosing by 20% [57]. Reconstruction with polyetheretherketone (PEEK) cages allowed for more consistent radiation dose delivery relative to titanium cages and polymethylmethacrylate (PMMA) based reconstruction [58]. Carbon fiber-reinforced PEEK (CFR-PEEK) corpectomy devices similarly decrease off-target radiation dosing (up to 68% versus titanium) while offer similar structural stiffness [59].

CFR-PEEK instrumentation has consequently garnered increased interest. It has similar fatigue tolerance to titanium [60], with lower imaging artifact [61] and less radiotherapy dose perturbation [62] Reduction in radiotherapy dose perturbation is even greater for proton and charged particle radiotherapy (up to 90%) [55,56,63,64]. However, CFR-PEEK implants are significantly more expensive than comparable metal implants, raising the issue of whether their cost is worth the improved radiographic follow-up. Using hybrid constructs using CFR-PEEK screws only at the tumor level (±1 segment above/below) or using CFR-PEEK only for the corpectomy defect/anterior column reconstruction may help balance the increased costs of CFR-PEEK against its benefits. CFR-PEEK instrumentation additionally cannot be contoured postmanufacture (curvatures are preset by the manufacturer) [65], limiting their utility for *in situ* deformity correction. Constructs seeking concomitant deformity correction may therefore employ titanium alloy rods with CFR-PEEK screws, offering some radiographic artifact suppression while still affording intraoperative deformity correction.

## Unique features of deformity in the oncology population

### Systemic therapy and radiotherapy

Unlike surgery for ASD, which seeks to “cure” the deformity, surgery for vertebral column neoplasms is seldom curative [66]. Noted exceptions are surgeries for primary bony malignancies, where *en bloc* resection with negative margins can improve overall survival [67,68]. Additionally, in select selected patients with isolated metastatic disease (usually from renal cell or thyroid primary lesions), *en* bloc resection can be considered as a strategy for improving overall survival [69]. However patients should have good cardiopulmonary reserve and well-controlled primary disease. In both primary and metastatic tumor cases; however, there is often an impetus to expedite initiation of radiotherapy and/or resumption of systemic therapy (metastatic disease) as this correlates with improved survival [70]. Classical chemotherapies [71,72] and anti-VEGF agents [73] impair wound healing by blocking cell proliferation and angiogenesis. Accordingly, they must generally be held 2 or more weeks perioperatively [74]. Surgical planning must therefore weigh the need for a more aggressive surgery that aims to correct any concomitant deformity against the need to expedite the resumption of systemic therapy.

Radiotherapy can similarly cause wound complications by killing the rapidly dividing cells involved wound healing and early scar formation. This side effect is reduced with focused photon beam modalities but remains a concern. Clinicians must therefore balance the improved local control afforded by early radiotherapy against the increased risk of wound complications. Various reviews [75] and survey-based studies [74,76] have suggested delaying radiotherapy 1 to 6 week after surgery but all are based on low-quality evidence. Evidence guiding radiotherapy initiation after surgery for primary malignancies is similarly week, though a position paper by several spinal oncology found most recommend waiting 3 to 8 weeks [77].

Of additional concern, high-dose radiation causes trabecular bone loss [78], which may weaken screw purchase [79], and radiation therapy appears to inhibit bony fusion [79] with a recent single-center series [80] noting only 29% of patients had any evidence of arthrodesis and 8.2% had complete fusion. Hardware failure was uncommon (4%) due to short overall survival, but the high nonunion rates suggest success in spine oncology may be measured in terms of successful immobilization and deformity correction, not fusion [81].

### Focal deformity

Deformity in tumors often results from focal kyphosis in the setting of a pathologic compression or burst fracture [82]. These deformities are generally mobile and associated with mechanical pain. In the absence of epidural disease, vertebral cement augmentation via vertebroplasty or kyphoplasty may be a reasonable option as both have been shown to offer significant improvement in pain [83,84]. Rates of segmental correction vary [82,85], though Kim and colleagues [86] reported an average of 11° of correction when kyphoplasty followed 2 days of postural fracture reduction. Long-term follow-up suggests that such corrections may not prove robust, with Lim et al. noting complete loss of correction by one-year [87]. Yet they may be optimal for patients with limited survival, offering symptom palliation without the morbidity of open surgery. Newer percutaneous strategies, such as the SpineJack (Stryker, Kalamazoo, MI) may offer superior radiographic correction to conventional cement-augmentation techniques; however, they are currently contraindicated in the setting of pathologic fracture. Consequently, for patients healthy enough for surgery, correction with pedicle screw instrumentation is likely the best option to correct underlying deformity.

Deformity can also be focal in primary pathologies (see Example Case 1), but this is generally deformity created by the surgeon during tumor resection [24]. Residual deformity correlates with both poorer PROs [14] and more mechanical complications, specifically implant failure and adjacent segment disease [24]. Reconstruction post-tumor delivery should therefore focus on preservation [and if necessary, restoration] of normal alignment.

### Multilevel disease

Nearly 40% of spinal metastasis patients have multilevel disease [88], which can lead to multifocal deformity. Multifocal disease also causes poor bone quality and screw purchase at multiple levels, which may in part be addressed using PMMA cement augmentation [89,90], especially where there is concomitant osteoporosis [91]. It may therefore allows surgeons to use shorter constructs [92] with accordingly smaller incisions and potentially faster recovery and resumption of systemic therapy. Fenestrated screws are a popular option for cement-augmentation as then can be used even during percutaneous fixation. However, traditional cement-augmentation with screw tract prefilling may afford greater pullout strength [93] as fenestrated screws deposit cement predominately at the screw tip [94] The former may therefore be preferrable for procedures seeking to correct deformity due to the forces required for intraoperative reduction (see Case Example 2). While augmentation with larger cement volumes may improve pullout strength [90] it is at the cost of increased cement extravasation risk and our tendency is to use 1 to 1.5 mL per screw for thoracic levels and 3 mL per screw for lumbar levels [89,95]; we do not employ cement augmentation at cervical levels.

### The need for radiographic follow-up

Spinal tumor patients generally require serial radiographic surveillance, which is negatively impacted by the artifact produced by metal implants [96] CFR-PEEK instrumentation lacks this artifact though offers limited intraoperative correction as rods cannot undergo *in situ* contouring [81] Biomechanically [60,97] it offers similar fatigue resistance [60], axial loading [97], and construct stiffness [98,99] with potentially smaller increases in adjacent segment motion and disc pressures with a resultant decrease in adjacent segment disease risk [99].

Where deformity correction is desired then, a hybrid construct may be optimal, blending the radiographic benefits of CFR-PEEK screws and interbodies with the cost benefits and *in situ* deformity correction afforded by titanium rods. Hybrid constructs often employ CFR-PEEK screws 1 to 2 levels immediately cranial and caudal to the tumor target with titanium screws elsewhere [81] CT phantom work [64] has shown this to significantly cut radiographic artifact and reduce radiographic planning uncertainty to a level approximating that seen with pure CFR-PEEK constructs.

### Anterior column reconstruction

Nearly 80% of vertebral column metastases affect the vertebral body [100] and primary bony malignancies benefitting from *en bloc* resection [67,68,101], also commonly localize to or involve the vertebral body. Spinal tumor treatment consequently often involves partial or complete corpectomy/spondylectomy and anterior column reconstruction. Reconstruction options include strut allograft, vascularized fibular strut autograft, PMMA cement, titanium mesh or expandable cages, and static or expandable CFR-PEEK cages [[102], [103], [104]]. Defect size, surgical approach, (posterior versus anterior; minimally invasive versus open), adjacent bone quality, and radiotherapy needs guide reconstruction choice. PMMA cement is one of the oldest techniques [104] and advantages include simplicity of use (requiring minimal carpentry relative to cage reconstruction), relatively low cost, and high cross-sectional area (offering minimal subsidence risk) [105] CFR-PEEK cages improve radiotherapy planning and off-target dosing relative to titanium cages [59], and better approximate the Young’s modulus of cancellous and cortical bone [106], potentially reducing potential subsidence risk. However, they are significantly more expensive than titanium cages. Strut grafts similarly allow for effective postoperative radiological monitoring but potentially altering biomechanics by shifting the instantaneous axis of rotation anteriorly [107], though this can be corrected with supplementary posterior instrumentation. Additionally, fibular strut grafts have narrow cross-sections that may increase subsidence risk and less capacity for bone graft material relative to nonexpandable cages [106] Vascularized fibular struts offer reasonable fusion outcomes, with complete union in up to 76% of cases following *en bloc* spondylectomy [103]. They may therefore be preferrable in the primary tumor population, where neoadjuvant or adjuvant radiotherapy often results in an environment that is extremely hostile to bone regeneration. However, the additional morbidity associated with vascularized autograft harvest unlikely justified in metastasis surgeries. In these patients, a more minimally invasive approach is often preferred and surgeons should consider if anterior reconstruction is needed or would be tolerated by the adjacent endplates. Separation surgery is popular in such cases [66], but it affords minimal opportunity for spinal realignment. Additionally, extended anterior column resection can improve local control [108] and can be achieved through a minimal access approach [109] to reduce procedural invasiveness. For these mini-open cases, reconstruction with an expandable cage is preferred as it minimizes device profile during placement while still affording substantial anterior column height restoration. Some also have self-adjusting end caps that maintain effective vertebral endplate-cage apposition throughout deformity correction [102]. This may reduce endplate stresses and subsidence postcorrection [110]. Both CFR-PEEK and titanium alloy devices with large endplates to minimize endplate stresses exist, though experiences with titanium-based devices are more extensive.

Corpectomy and *en bloc* spondylectomy are both highly destabilizing and anterior column reconstruction alone is generally insufficient. Construct design must balance the surgical morbidity and associated artifact of increased instrumentation against the benefits of increased stability. Long-segment instrumentation (±3 segments from the corpectomy level) offers the greatest stability and is superior to 1-2-segment instrumentation [111,112]. It also decreases endplate stresses following multilevel corpectomy [112], though this may also lead to stress shielding and nonunion.

Multirod constructs are popular in ASD surgery, especially at the lumbosacral junction and pedicle subtraction osteotomy (PSO) sites, as the second rod increases construct stability and decreases primary rod strain. Multiple strategies for secondary rod integration have been described, but the use of an accessory rod—one connected to the primary rod—is common. However, such accessory rods may not decrease primary rod strain [113,114], whereas secondary rods directly anchored to the spine reduce primary rod stresses significantly [113] Offset connectors are not presently marketed for CFR-PEEK rods, so surgeons opting for multirod constructs may have to utilize titanium alloy rods or dual-rod constructs in which both rods have direct pedicle fixation. For cervical lesions, the placement of a tension-band plate as part of the anterior approach offers greater stability than the addition of extra posterior rods [115] Last, cross-connectors do not decrease stresses on the corpectomy cage, but increase rod stain [116], and therefore may increase hardware failure risk. Additionally, the artifact created by the cross-connector further complicates radiographic surveillance and radiotherapy planning.

There is a relative paucity of literature comparing outcomes between spinal metastasis patients who undergo corpectomy and those treated with posterolateral instrumented fusion alone. While corpectomy offers the greatest opportunity for deformity correction, cement-augmented instrumented fusion may offer sufficient correction in many oncologic patients and further work is needed to compare local control, deformity correction, pain palliation, and PROs between these strategies.

### Pelvic reconstruction

Lumbopelvic reconstruction following sacrectomy is one of the biomechanically most complex operations faced by spinal oncologists owing to the high stresses across the lumbosacral junction [117] and the potential for poor realignment to create significant sagittal plane deformity. With removal of the sacrum, the patient ostensibly loses their innate pelvic incidence; the new PI is dictated by the relative curvature of the rods linking the bony pelvis to the lumbar spine [118] Dual rod instrumentation with dual pelvic anchors bilaterally provides the most stable pelvic reconstruction [[119], [120], [121]] and 4 rod constructs have the lowest risk of mechanical complications following sacrectomy clinically [122]. Use of a transiliac bar does not appear to afford additional stability [119] and in our hands, an upper instrumented vertebra of L3 is preferrable to have multiple points of fixation above the sacrectomy defect, especially in older patients with poor bone quality. Long pelvic screws passing ventral to the lumbosacral pivot point offer the greatest resistance against the large angular moments applied at the lumbosacral junction [123,124]. The sacral defect can be reconstructed with an interiliac femur strut but this appears inferior to other options, including bilateral L5-iliac cage struts or an expandable interbody device secured onto a transiliac bar for S1 body replacement [125]. For dual pelvic fixation we favor using intraoperative navigation to allow for stacked S2AI screw placement [126].

### Fusion

Rates of fusion in spine tumor patients are overall low [80] perhaps owing to impaired physical reserve and increased rates of malnutrition [127], which may lead to poor wound and bone healing. Radiotherapy also inhibits bony fusion. Reconstruction with vascularized autograft can improve fusion outcomes [103], yet the associated morbidity is likely justified only in primary tumors. Consequently, the optimal target for most spine tumor patients may not be successful fusion but rather long-term restoration or maintenance of spinal alignment.

## Synthesis

**Fig. 2:**
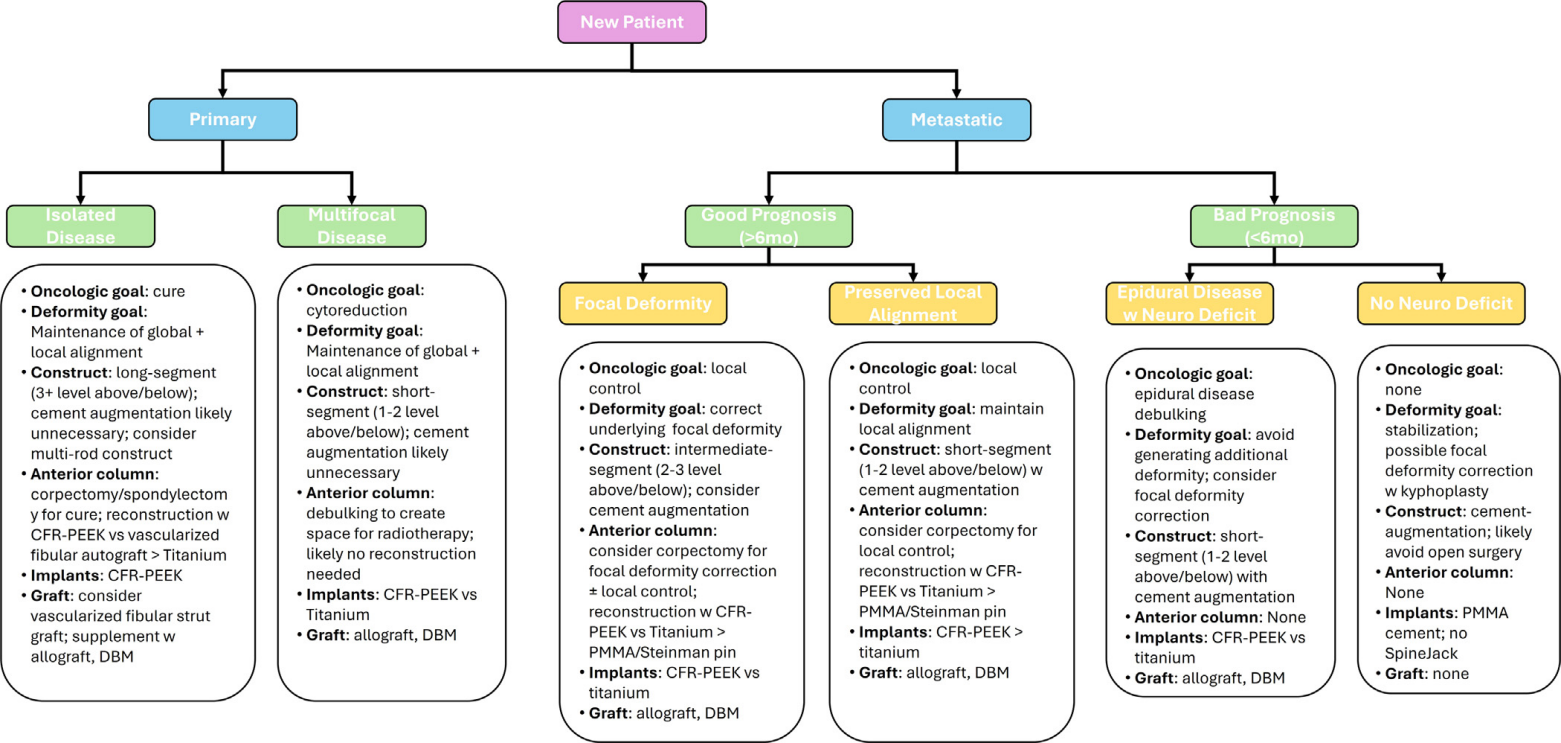
Flowsheet for suggested approach to oncologic and deformity considerations in patients undergoing treatment for vertebral column tumors. In general, patients being treated for primary lesions may be considered for more aggressive corrective surgeries given the attendant morbidity of surgery is already high. However, they infrequently present with focal deformity. Patients with metastases often benefit from a more reserved approach to deformity correction, one that seeks to correct focal deformity in patients with good prognoses. Those with poor overall prognoses may not derive enough benefit from deformity correction to undergo open surgery in the absence of other indications (eg, epidural compression).

The present review, while not all encompassing, attempts to touch on important aspects of spinal deformity in spine tumor patients; we highlight a general approach to management in Fig. 2. For isolated primary lesions realignemtn can be far more aggressive than for patients with diffuse metastatic disease. For example, in a patient presenting with a lumbar chordoma with no metastatic disease, the oncologic goal of surgery is cure, which can potentially be achieved with *en bloc* spondylectomy. This 3-column osteotomy is highly destabilizing but also affords surgeons the opportunity to correct underlying global and focal deformity. While it is clear the construct should be selected to address any focal deformity, the degree to which global deformity should be corrected may be debated. Our preference is to aim for a normative sagittal alignment (SVA <5 cm; PI-LL mismatch <10°) as this appears to correlate with better patient physical function and lower rates of mechanical complications. However, given that high-dose neoadjuvant/adjuvant radiotherapy will likely be given, the construct must offer significant stability given the high odds of nonunion. We prefer a long-segment (±3 levels), multirod construct, taking care to avoid terminating the construct at a junctional level. CFR-PEEK screws/rods are preferrable owing to the need for long-term radiographic surveillance and radiotherapy delivery. However, if there is significant baseline focal deformity, we opt for titanium rods, which allow for *in situ* deformity correction. In all cases oncologic cure should be prioritized over deformity correction, but where there is noted underlying focal deformity, we favor correction of the underlying deformity over optimal radiographic surveillance. Use of a vascularized fibular autograft should be considered to help maximize the odds of successful fusion, though as mentioned above, stable nonunion with preserved alignment may be a more realistic long-term goal.

By contrast, in a patient with diffuse metastases, the decision to pursue surgery hinges on the presence of epidural disease. For those with epidural disease, separation surgery should be considered, and use of cement-augmented pedicle screws may allow for some focal deformity correction. These patients commonly are malnourished and frail, so we favor more minimally invasive approaches and minimize the use of long- and multirod constructs. In these frail patients, a surgical strategy that employs less invasive surgical techniques to prevent further deformity may be preferrable to a more aggressive strategy that corrects the deformity but with higher attendant morbidity. An even less invasive approach is favored for those without epidural disease; surgery for deformity correction alone is likely counterproductive given the high morbidity in a patient with poor expected survival. Percutaneous stabilization via vertebroplasty or kyphoplasty may therefore be a better option.

For patients with recurrent primary tumors or oligometastatic disease from nonspine primary lesions, the relative weight of deformity and oncologic considerations falls somewhere in the middle. In general, primacy is given to addressing neural element compression, as patient function is the priority. In those with metastatic disease, cure is impossible, and so greater weight is given to correction of local deformity. In patients with very good functional status and healthy adjacent vertebrae with good bone quality, we consider corpectomy to improve local control and facilitate global deformity correction. Cement augmentation should be considered to improve screw-bone purchase and facilitate correction of the deformity. Both titanium and CFR-PEEK implants are reasonable. Similarly, for patients with recurrent primary tumors, local spread is likely. Radiographic surveillance is generally of greater import though and so we would favor CFR-PEEK implants given the lower radiographic artifact. Given that surgery will likely attempt maximally safe cytoreduction, anterior reconstruction may be required. This offers the opportunity for greater local and global deformity correction, but we favor a multirod construct for long-term stability. Vascularized fibular strut autograft can be considered, but such a strategy may not be merited in a patient likely to have further recurrence and the need for additional cytoreductive surgery [128].

### Case 1

**Fig. 3:**
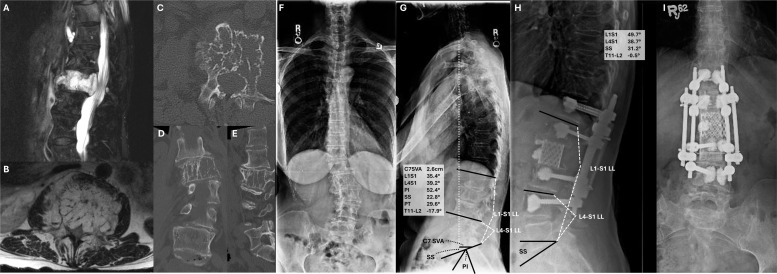
Case 1. Patient presented with mechanical low back pain and neurogenic pseudoclaudication. MR showed A) a T2-hyperintense, B) homogeneously contrast-enhancing lesion of the L2 body with focal collapse and bony retropulsion, causing spinal canal stenosis. C–E) CT demonstrated extensive osteolysis in all planes. F, G) Upright full-length scoliosis films demonstrated a mild levoscoliosis with relative preservation of sagittal balance but a mild reversal of lordosis at the thoracolumbar junction. H, I) Postoperative upright radiographs show reconstruction with a T12-L4 instrumented fusion with titanium cage reconstruction in the L2 corpectomy defect.

A 67-year-old woman presented with mechanical back pain localizing to the upper lumbar spine. She also endorsed progressive bilateral radicular leg pain worse with ambulation and consistent with neurogenic pseudoclaudication. Imaging (Fig. 3) demonstrated a T2-hyperintense, avidly contrast-enhancing lesion of the L2 body with <50% height loss and osteolysis of the left pedicle (SINS), with an accompanying mild focal kyphoscoliosis at the thoracolumbar junction. Biopsy demonstrated aggressive hemangioma. Given the overall preserved sagittal balance, the decision was made to use an intermediate-length construct with an L2 corpectomy to remove the tumor; the defect was reconstructed with a titanium mesh cage. Posterolaterally a dual-rod construct was employed, along with cement-augmentation of the screws to improve purchase and fibular strut allograft secured to the rods with cables to serve as osteoconductive elements for long-term fusion. Titanium implants were used given the oncologically benign process and absence of a need for serial MR follow-up.

### Case 2

**Fig. 4:**
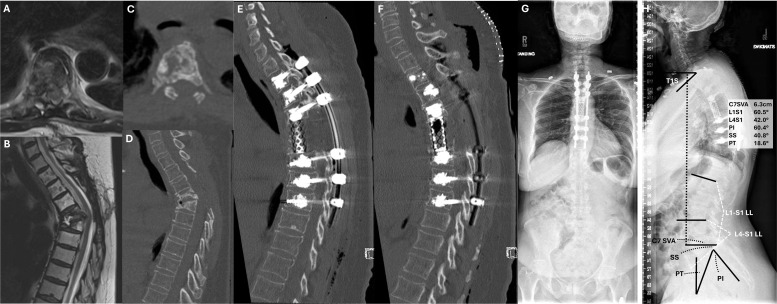
Case 2. Patient with previous T3-T5 laminectomy who subsequently presented to clinic with signs and symptoms of lower extrmity myelopathy. A,B) MR and C,D) demonstrated focal kyphosis at the T5-6 levels with >50% vertebral body collapse and draping or bowstringing of the spinal cord over the kyphotic bodies. E, F) The patient underwent T5-6 corpectomy with titanium mesh cage reconstruction and long-segment instrumented fusion from T2 to T9. G, H) Postoperative upright radiographic films showed elimination of the kyphotic defect with good overall sagittal and coronal balance.

A woman in her mid-60 s with known history of multiple myeloma presented to the emergency department with acute onset paraparesis. Imaging demonstrated high-grade epidural spinal cord compression, for which she underwent T3-T5 open laminectomy with debulking of epidural disease followed by adjuvant radiotherapy. The patient recovered lower extremity function and initially did well but subsequently returned to clinic with upper back pain and increased gait imbalance; she had a spastic gait consistent with thoracic myelopathy on exam. Imaging (Fig. 4) showed >50% collapse of the T5 and T6 vertebral bodies with focal kyphosis of 36.9° causing bowstringing of the spinal cord. The patient had well-controlled systemic disease and was otherwise healthy, so the decision was made to correct the underlying deformity. She underwent a single stage procedure with T5-6 corpectomy and anterior column reconstruction with a titanium mesh cage. Posterolateral fusion was performed with a long-segment construct (T2-T9) and morselized allograft. Cement-augmentation was used to improve screw purchase and facilitate intraoperative deformity correction, especially given the patients history of myeloma and concern for poor underlying bone quality. Postoperative upright radiographs showed good correction of the focal deformity and restoration of a relatively normal global balance. While her SVA was above 5 cm (6.3 cm), is was more consistent with the age-normative target recommended by Lafage et al. [129] based upon ODI scores in adult spinal deformity patients. She also had minimal lumbopelvic mismatch (LL 61° vs PI 60°) and showed no pelvic retroversion (19°) to suggest compensation for persistent deformity. Titanium implants were selected so as to improve long-term surveillance relative to cobalt-chrome implants; CFR-PEEK implants were not widely available at the time of her surgery.

## Conclusion

Given improvements in survival following diagnosis with spinal malignancy, there is increasing attention being paid to the impact of poor alignment on postoperative outcomes in the spinal oncology population. This review focuses on the application of deformity principles to the spinal oncology patient, discussing the correlation of deformity with patient-reported outcomes, the evaluation of underlying bone quality, instrumentation selection, and bony defect reconstruction. While the present review cannot be all encompassing, we hope that it will serve as an effective primer for those treating patients with bony spine tumors.

## IRB

Approval: IRB approval was not required for the present narrative review as it did not meet the definition of human subjects research. ronal balance.

## Funding

None.

## Declaration of competing interest

None.
